# Insecticide resistance in malaria vectors along the Thailand-Myanmar border

**DOI:** 10.1186/s13071-017-2102-z

**Published:** 2017-03-31

**Authors:** Victor Chaumeau, Dominique Cerqueira, John Zadrozny, Praphan Kittiphanakun, Chiara Andolina, Theeraphap Chareonviriyaphap, François Nosten, Vincent Corbel

**Affiliations:** 1 Centre hospitalier universitaire de Montpellier, Montpellier, France; 2 Maladies Infectieuses et Vecteurs, Ecologie, Génétique, Evolution et Contrôle, Institut de Recherche pour le Développement, Montpellier, France; 3 Shoklo Malaria Research Unit, Mahidol-Oxford Tropical Medicine Research Unit, Faculty of Tropical Medicine, Mahidol University, Mae Sot, Thailand; 4 Department of Entomology, Faculty of Agriculture, Kasetsart University, Bangkok, Thailand; 5 Centre for Tropical Medicine and Global Health, Nuffield Department of Medicine, University of Oxford, Oxford, UK

**Keywords:** Malaria, Thailand-Myanmar border, *Anopheles*, Pyrethroids, Insecticide resistance, kdr mutation, Southeast Asia

## Abstract

**Background:**

There is a paucity of data about the susceptibility status of malaria vectors to Public Health insecticides along the Thailand-Myanmar border. This lack of data is a limitation to guide malaria vector-control in this region. The aim of this study was to assess the susceptibility status of malaria vectors to deltamethrin, permethrin and DDT and to validate a simple molecular assay for the detection of knock-down resistance (kdr) mutations in the study area.

**Methods:**

*Anopheles* mosquitoes were collected in four sentinel villages during August and November 2014 and July 2015 using human landing catch and cow bait collection methods. WHO susceptibility tests were carried out to measure the mortality and knock-down rates of female mosquitoes to deltamethrin (0.05%), permethrin (0.75%) and DDT (4%). DNA sequencing of a fragment of the voltage-gated sodium channel gene was carried out to identify knock-down resistance (kdr) mutations at position 1014 in mosquitoes surviving exposure to insecticides.

**Results:**

A total of 6295 *Anopheles* belonging to ten different species were bioassayed. Resistance or suspected resistance to pyrethroids was detected in *An. barbirostris* (*s.l*.) (72 and 84% mortality to deltamethrin (*n* = 504) and permethrin (*n* = 493) respectively), *An. hyrcanus* (*s.l*.) (33 and 48% mortality to deltamethrin (*n* = 172) and permethrin (n = 154), respectively), *An. jamesii* (87% mortality to deltamethrin, *n* = 111), *An. maculatus* (*s.l*.) (85 and 97% mortality to deltamethrin (*n* = 280) and permethrin (*n* = 264), respectively), *An. minimus* (*s.l*.) (92% mortality, *n* = 370) and *An. vagus* (75 and 95% mortality to deltamethrin (*n* =148) and permethrin (*n* = 178), respectively). Resistance or suspected resistance to DDT was detected in *An. barbirostris* (*s.l*.) (74% mortality, *n* = 435), *An. hyrcanus* (*s.l*.) (57% mortality, *n* = 91) and *An. vagus* (97% mortality, *n* = 133). The L1014S kdr mutation at both heterozygous and homozygous state was detected only in *An. peditaeniatus* (Hyrcanus Group).

**Conclusion:**

Resistance to pyrethroids is present along the Thailand-Myanmar border, and it represents a threat for malaria vector control. Further investigations are needed to better understand the molecular basis of insecticide resistance in malaria vectors in this area.

## Background

Vector-borne diseases account for 17% of all infectious diseases, causing more than 1 million deaths annually [1]. Malaria causes more than 600,000 deaths every year, mostly in children under 5 years [2]. Vector-control is an essential component of malaria control, and it relies mainly on long-lasting insecticidal nets (LLINs) and indoor residual spraying (IRS) [3]. In low transmission settings, especially where residual transmission (i.e. transmission that is not prevented by LLINs and IRS) is prominent, LLINs and IRS must be supplemented by other methods to target early feeding, exophagic and zoophagic malaria vectors [4].

Pyrethroids are widely used for vector control because of their strong insecticidal effect and their low toxicity to mammals [5]. They induce a rapid “knock-down” (KD), and they have irritant and excito-repellent properties against susceptible mosquitoes [6]. Sadly, pyrethroid resistance in malaria vectors has been identified in at least 64 countries [5] and is spreading worldwide [7] because of the intense selection pressure caused by agricultural practices [8] and the large-scale implementation of malaria vector control interventions [7]. Resistance has been implicated in the reduced efficacy of vector control interventions such as IRS and LLIN [9, 10] and malaria resurgence [11–17]. Routine monitoring of resistance and detection of temporal changes in both prevalence and intensity of resistance are needed to guide malaria vector interventions and resistance management plan [18].

Compared to Africa, there is a notable lack of data on insecticide resistance in Southeast Asian malaria vectors. Previous report of resistance in malaria vectors from the GMS are summarised in Table 1. Resistance to several pyrethroids and decreased susceptibility to DDT were described for *An. minimus* (*s.s*.) in Vietnam, Thailand and Cambodia [19, 20]. Resistance to organophosphate was reported in *An. maculatus* and *An. sawadwongporni* in northern Thailand [21]. Suspected resistance to pyrethroids (Vietnam) and DDT (Cambodia) was described in *An. dirus* (*s.s*.) [19] and suspected resistance to DDT and fenitrothion were reported in laboratory reared *An. cracens* from Thailand and Malaysia [22]. High levels of resistance to DDT and permethrin were reported in *An. vagus* in the Greater Mekong Subregion (GMS) [19, 23].

**Table 1:** Insecticide resistance in malaria vectors from the Greater Mekong Subregion

Species	Insecticide	Site	Date	Reference
*An. minimus* (*s.s*.)	DDT	Thailand, Vietnam, Cambodia	1999, 2003, 2008	[19, 20, 52]
	Pyrethroids^a^	Vietnam	2008	[19]
*An. maculatus* (*s.s*.) and *An. sawadwongporni*	DDT	Thailand	1999	[52]
	Methylparathion	Thailand	2005	[21]
*An. dirus* (*s.s*.)	DDT	Cambodia	2008	[19]
	Pyrethroids^b^	Vietnam	2008	[19]
*An. cracens*	DDT, fenitrothion	Thailand and Malaysia	1984	[22]
*An. vagus*	DDT, pyrethroids^c^	Thailand, Vietnam, Cambodia	2008	[19]

^a^ alpha-cypermethrin, lambda-cyhalothrin and permethrin

^b^ alpha-cypermethrin and lambda-cyhalothrin

^c^ alpha-cypermethrin, deltamethrin, lambda-cyhalothrin and permethrin

The mechanisms of insecticide resistance in mosquitoes are multiple and include behavioural and physiological changes leading to insecticide avoidance, reduced penetration, sequestration, target site modification (knock-down resistance or kdr mutation for pyrethroids and DDT) and increased biodegradation [13, 24, 25]. With the exception of *An. sinensis* [26–30], resistance mechanisms remain largely unknown in Southeast Asian malaria vectors. In this region, metabolic resistance is thought to play a major role compared to target site mutations [27, 31]. Only a few studies have been conducted to understand the pathways involved in metabolic resistance, and they were limited to a laboratory adapted deltamethrin-resistant strain of *An. minimus* (*s.s*.) from Thailand [32–34]. Kdr mutations were reported in several anopheline species [23, 35] but were not detected in the major malaria vectors in the GMS [31].

The aim of the present study is to investigate the susceptibility status of malaria vectors to Public Health insecticides (deltamethrin, permethrin and DDT) along the Thailand-Myanmar border (TMB), and to assess the presence of kdr mutations [35]. Resistance data are deemed important to design more effective vector control interventions in the area.

## Methods

### Study sites and mosquito collection

**Fig. 1:**
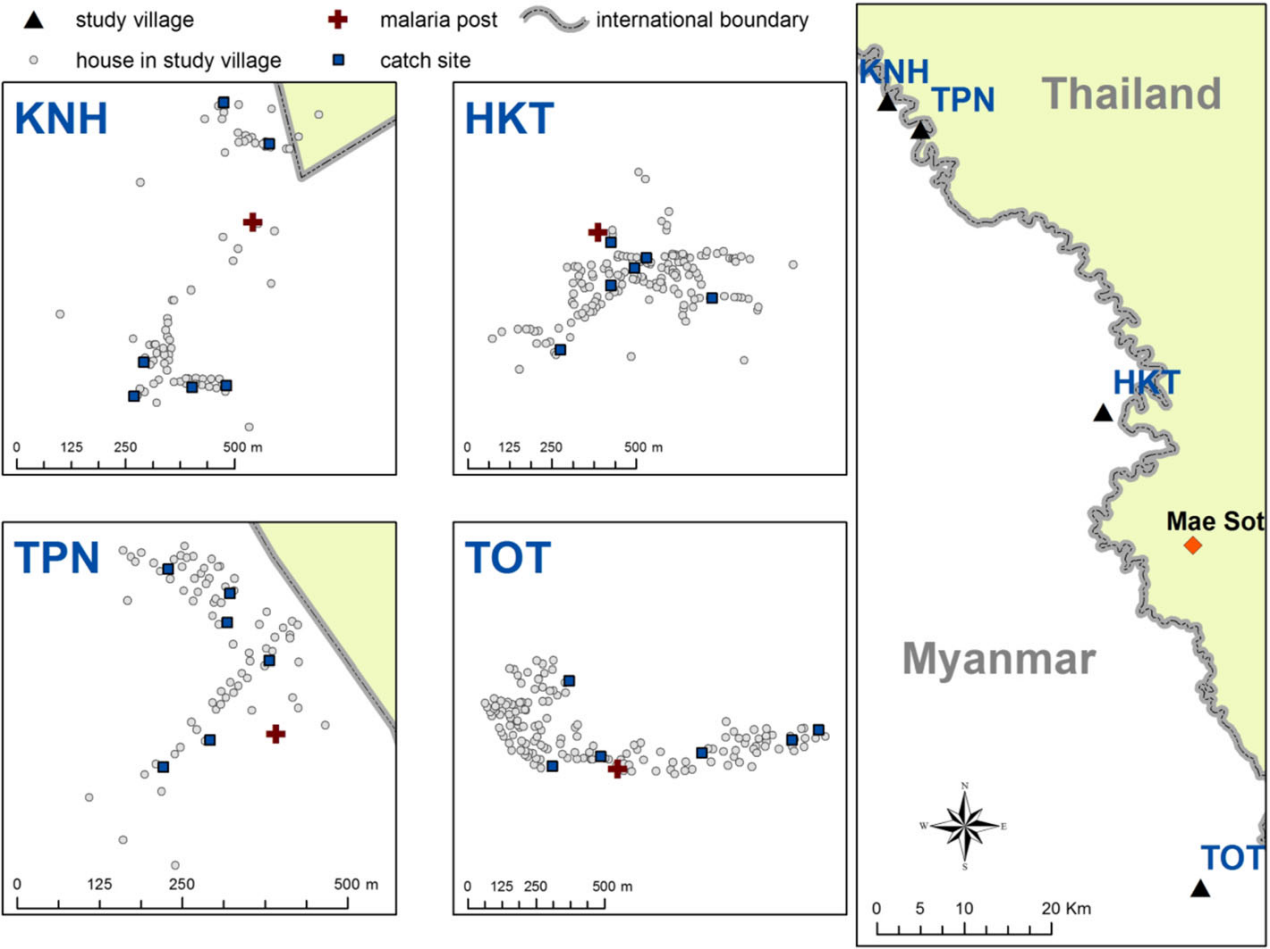
Map of the study sites. Three entomological surveys were conducted in four villages situated on the Myanmar side of the Thai-Myanmar border (HKT, Htee Kaw Taw; KNH, Ka Nu Hta; TPN, Htoo Pyin Nyar; and TOT, Tar Au Ta). Mosquitoes were collected using indoor and outdoor Human Landing Catch (HLC) in five sites and using Cow Bait Collection (CBC) in one site. Mosquitoes were shipped daily at the Shoklo Malaria Research Unit (SMRU, Mae Sot, Thailand) for identification, rearing and bioassays

Mosquito collections were carried out in four Karen villages located on the Myanmar side of the TMB, namely Htoo Pyin Nyar (TPN, 17°14'N, 98°29'E), Tar Au Ta (TOT, 16°36'N, 98°57'E), Ka Nu Hta (KNH, 17°18'N, 98°24'E) and Htee Kaw Taw (HKT, 16°85'N, 98°47'E) (Fig. 1). Three entomological surveys were conducted in August and November 2014 and July 2015 using human landing catch (HLC) and cow-bait collection (CBC) methods. Mosquitoes were collected individually using 5 ml plastic tubes and shipped daily to the Shoklo Malaria Research Unit (Mae Sot, Thailand). *Anopheles* were individually transferred to a clean and transparent plastic tube while still alive and identified by morphology [36]. The mosquitoes belonging to different species were separated into different cups and reared for 1–4 days until enough specimens were collected to run the bioassay. Mosquitoes collected by HLC and CBC were pooled together to increase the sample size.

### Susceptibility tests

Susceptibility tests were performed following the WHO guidelines for insecticide resistance monitoring in malaria vectors [37]. All materials used in this study were provided by the Vector Control Research Unit (VCRU), Universiti Sains Malaysia. Wild caught female Anopheles were exposed for 60 min to papers impregnated with deltamethrin (0.05%), permethrin (0.75%) or DDT (4%). The susceptibility status of two Anopheles laboratory strains [*An. minmus* (*s.s*.) from Kasetsart University, Bangkok, Thailand and *An. scanloni*, Shoklo Malaria Research Unit, Mae Sot, Thailand] were also determined (50 mosquitoes for each insecticide and the control). The number of knocked-down (KD) mosquitoes was recorded every 5 min for 60 min. The females were then transferred into holding tubes, provided with a sugar solution (10%), and kept at 26 °C with a relative humidity of 80%. Mortality was recorded after a 24 h observation period. Mosquitoes exposed for 1 h to paper impregnated with the carrier (silicone oil mixed with acetone) were used as controls. Tests were replicated when a sufficient number of specimens were collected. Results were interpreted as per WHO guidelines: confirmed resistance (mortality below 90%), suspected resistance (mortality between 90 and 98%) and susceptible (mortality over 98%) [37].

### kdr mutation detection

DNA was extracted from a leg of each specimen as previously described [38]. Amplification of a 300 bp segment of the voltage-gated sodium channel (VGSC) gene flanking the 1014 position was performed using the primer pair Ag-F kdr (5'-GAC CAT GAT CTG CCA AGA TGG AAT-3') and An-kdr-R2 (5'-GAG GAT GAA CCG AAA TTG GAC-3') described by Syaffrudin et al. [35]. The PCR mix was composed of 1 unit of Tfi DNA polymerase (Invitrogen™, Carlsbad, United States), 200 μM of dNTP mix (Invitrogen) which corresponded to 200 μM of each dNTP, 1.5 mM of MgCl2 (Invitrogen), and 400 μM of each primer. The PCR was conducted in a total reaction volume of 50 μl (3 μl of DNA template and 47 μl of PCR mix). The thermocycling protocol consisted in a first cycle of 5 min at 94 °C then 30 s at 45 °C, followed by 29 cycles of 30 s at 94 °C, 30 s at 50 °C and 1 min at 72 °C. The PCR product was sequenced by Macrogen™ (Seoul, South-Korea) using both primers. Each sequence was checked and cleaned manually using the Bioedit software version 7.1.9 (http://www.mbio.ncsu.edu//BioEdit/bioedit.html). A consensus sequence was generated for each specimen using the CAPS3 sequence assembly program [39] and then aligned using the Clustal Omega multiple sequence alignment program [40–42] (GenBank accession numbers KY677707–KY677716).

### Molecular identification of Anopheles by ITS2 sequencing

Amplification of the ITS2 was performed using the primer pair ITS2A (5'-TGT GAA CTG CAG GAC ACA T-3') and ITS2B (5'-ATG CTT AAA TTY AGG GGG T-3') described by Beebe et al. [43]. The PCR mix was composed of 1 unit of Goldstar DNA polymerase (Eurogentec™, Seraing, Belgium) and 400 μM of each primer. The PCR was conducted in a total reaction volume of 25 μl (4 μl of DNA template and 21 μl of PCR mix). The thermocycling protocol consisted in an initial activation step of 1 min at 94 °C, followed by 40 amplifcation cycles of 20 s at 94 °C, 20 s at 51 °C and 30 s at 72 °C, and a final elongation step of 30 s at 72 °C. The PCR product was sequenced by Macrogen™ (Seoul, South-Korea) using the ITS2A primer. *Anopheles* species was determined using the blastn algorithm of the online BLAST™ software [44] (accession numbers KY677698–KY677706).

### Data analysis

Adult mortality rate was corrected by the formula of Abbott [45] in the case of mortality > 5% in the control. Tests with > 20% mortality in the control were excluded from the analysis. Knock-down time 50 (KDT50) was determined by the log-probit method described by Finney [46] using R software [47]. The R code used to determine the knock-down time 50 (KDT50) and its confidence interval were adapted from Johnson et al. 2013 [48].

## Results

### Bioassays

In total, 5896 *Anopheles* (belonging to 9 groups of species) were collected in the villages and used to assess the insecticidal activity of deltamethrin 0.05% (*n* = 1,805), permethrin 0.75% (*n* = 1483) and DDT 4% (*n* = 1202) (Tables 2, 3 and 4). A total of 1406 specimens were used for the control (Tables 2, 3 and 4). Overall, the mean mortality in the control batch was 8.4% (119/1406), 3 of 46 tests were excluded from the analysis because the control mortality was > 20% (91/5896 specimens).

**Table 2:** Summary results of the bioassays with deltamethrin 0.05%

Taxa	*N* ^a^	% Mortality^b^	% KD^c^	KDT50^d^	Status^e^
*An. scanloni* (laboratory strain)	50	100 (na)	100 (na)	19.6 (18.4–20.7)	S
*An. minimus* (*s.s*.) (laboratory strain)	50	100 (na)	100 (na)	10.4 (9.4–11.4)	S
*An. annularis* (*s.l*.)	40	100 (na)	100 (na)	14.4 (13.1–15.6)	S
*An. barbirostris* (*s.l*.)	504	72 (68–76)	85 (82–88)	30.5 (28.7–32.4)	R
*An. hyrcanus* (*s.l*.)	172	33 (26–40)	27 (20–33)	131.3 (83–378.1)	R
*An. jamesii* (*s.l*.)	111	87 (81–94)	97 (94–100)	14 (11.7–16.2)	R
*An. kochi*	43	98 (93–100)	100 (na)	14.6 (13–16)	S
*An. maculatus* (*s.l*.)	280	85 (81–89)	89 (85–93)	18.8 (15.8–21.6)	R
*An. minimus* (*s.l*.)	370	92 (89–94)	99 (98–100)	15.4 (14.3–16.5)	SR
*An. tessellatus*	83	98 (94–100)	99 (96–100)	17.7 (16.6–18.8)	S
*An. vagus*	148	75 (68–82)	95 (91–98)	21.3 (19–23.4)	R

^a^ *N*: number of mosquito phenotyped

^b^ % Mortality: mortality rate (expressed in %) after 1 h of exposure to insecticide, recorded following a 24 h observation period; the values between parentheses indicate the 95% confidence interval of the mean mortality rate

^c^ % KD: rate of mosquitoes “knocked down” (KD, expressed in %) recorded after 1 h of exposure to insecticide; the values between parentheses indicate the 95% confidence interval of the mean KD rate

^d^ TKD50: time (expressed in minutes) necessary to “knock down” 50% of the mosquitoes; the values between parentheses indicate the 95% confidence interval of the TDKD50

^e^ Status: resistance status as defined by WHO [37]. Briefly, a mortality in the range 98–100% indicates susceptibility; a mortality between 90 and 97% indicates suspected resistance; a mortality < 90% indicates confirmed resistance as long as 100 specimens have been phenotyped

*Abbreviations*: *na*, not applicable; *R*, resistant; *S*, suceptible; *SR*, suspected resistance

**Table 3:** Summary results of the bioassays with permethrin 0.75%

Taxa	*N* ^a^	% Mortality^b^	% KD^c^	KDT50^d^	Status^e^
*An. scanloni* (laboratory strain)	50	100 (na)	98 (94–100)	25.6 (24.2–27)	S
*An. minimus* (*s.s*.) (laboratory strain)	50	100 (na)	100 (na)	13.5 (11.5–15.3)	S
*An. annularis* (*s.l*.)	0	na	na	na	na
*An. barbirostris* (*s.l*.)	493	84 (81–88)	90 (88–93)	26.6 (25.8–27.5)	R
*An. hyrcanus* (*s.l*.)	154	48 (40–56)	32 (24–39)	116.2 (83–212.1)	R
*An. jamesii* (*s.l*.)	54	98 (95–100)	100 (na)	11.3 (10.3–12.2)	S
*An. kochi*	0	na	na	na	na
*An. maculatus* (*s.l*.)	264	97 (95–99)	97 (95–99)	16.7 (15.6–17.8)	SR
*An. minimus* (*s.l*.)	340	98 (96–99)	99 (98–100)	15 (14.4–15.6)	S
*An. tessellatus*	0	na	na	na	na
*An. vagus*	178	95 (92–98)	100 (na)	15.3 (14.3–16.3)	SR

^a^ *N*: number of mosquito

^b^ % Mortality: mortality rate (expressed in %) after 1 h of exposure to insecticide, recorded following a 24 h observation period; the values between parentheses indicate the 95% confidence interval of the mean mortality rate

^c^ % KD: rate of mosquitoes “knocked down” (KD, expressed in %) recorded after 1 h of exposure to insecticide; the values between parentheses indicate the 95% confidence interval of the mean KD rate

^d^ TKD50: time (expressed in minutes) necessary to “knock down” 50% of the mosquitoes; the values between parentheses indicate the 95% confidence interval of the TDKD50

^e^ Status: resistance status as defined by WHO [37]. Briefly, a mortality in the range 98–100% indicates susceptibility; a mortality between 90 and 97% indicates suspected resistance; a mortality < 90% indicates confirmed resistance as long as 100 specimens have been phenotyped

*Abbreviations*: *na*, not applicable; *R*, resistant; *S*, suceptible; *SR*, suspected resistance

**Table 4:** Summary results of the bioassays with DDT 4%

Taxa	*N* ^a^	% Mortality^b^	% KD^c^	KDT50^d^	Status^e^
*An. scanloni* (laboratory strain)	50	84 (74–94)	42 (28–56)	63.2 (59.8–69.7)	S
*An. minimus* (*s.s*.) (laboratory strain)	50	100 (na)	98 (94–100)	38.3 (36.9–39.7)	R
*An. annularis* (*s.l*.)	0	na	na	na	na
*An. barbirostris* (*s.l*.)	435	74 (70–78)	49 (45–54)	61 (59.1–63.2)	R
*An. hyrcanus* (*s.l*.)	91	57 (47–67)	30 (20–39)	84.2 (70.9–112.1)	R
*An. jamesii* (*s.l*.)	59	98 (95–100)	88 (80–96)	33.3 (31.3–35.2)	S
*An. kochi*	0	na	na	na	na
*An. maculatus* (*s.l*.)	239	99 (97–100)	9 (94–99)	25.5 (23.4–27.4)	S
*An. minimus* (*s.l*.)	245	100 (na)	100 (na)	23.4 (21.8–24.9)	S
*An. tessellatus*	0	na	na	na	na
*An. vagus*	133	97 (94–100)	91 (86–96)	35.3 (34–36.6)	SR

^a^ *N*: number of mosquito phenotyped

^b^ % Mortality: mortality rate (expressed in %) after 1 h of exposure to insecticide, recorded following a 24 h observation period; the values between parentheses indicate the 95% confidence interval of the mean mortality rate

^c^ % KD: rate of mosquitoes “knocked down” (KD, expressed in %) recorded after 1 h of exposure to insecticide; the values between parentheses indicate the 95% confidence interval of the mean KD rate

^d^ TKD50: time (expressed in minutes) necessary to “knock down” 50% of the mosquitoes; the values between parentheses indicate the 95% confidence interval of the TDKD50

^e^ Status: resistance status as defined by WHO [37]. Briefly, a mortality in the range 98–100% indicates susceptibility; a mortality between 90 and 97% indicates suspected resistance; a mortality < 90% indicates confirmed resistance as long as 100 specimens have been phenotyped

*Abbreviations*: *na*, not applicable; *R*, resistant; *S*, suceptible; *SR*, suspected resistance

**Fig. 2:**
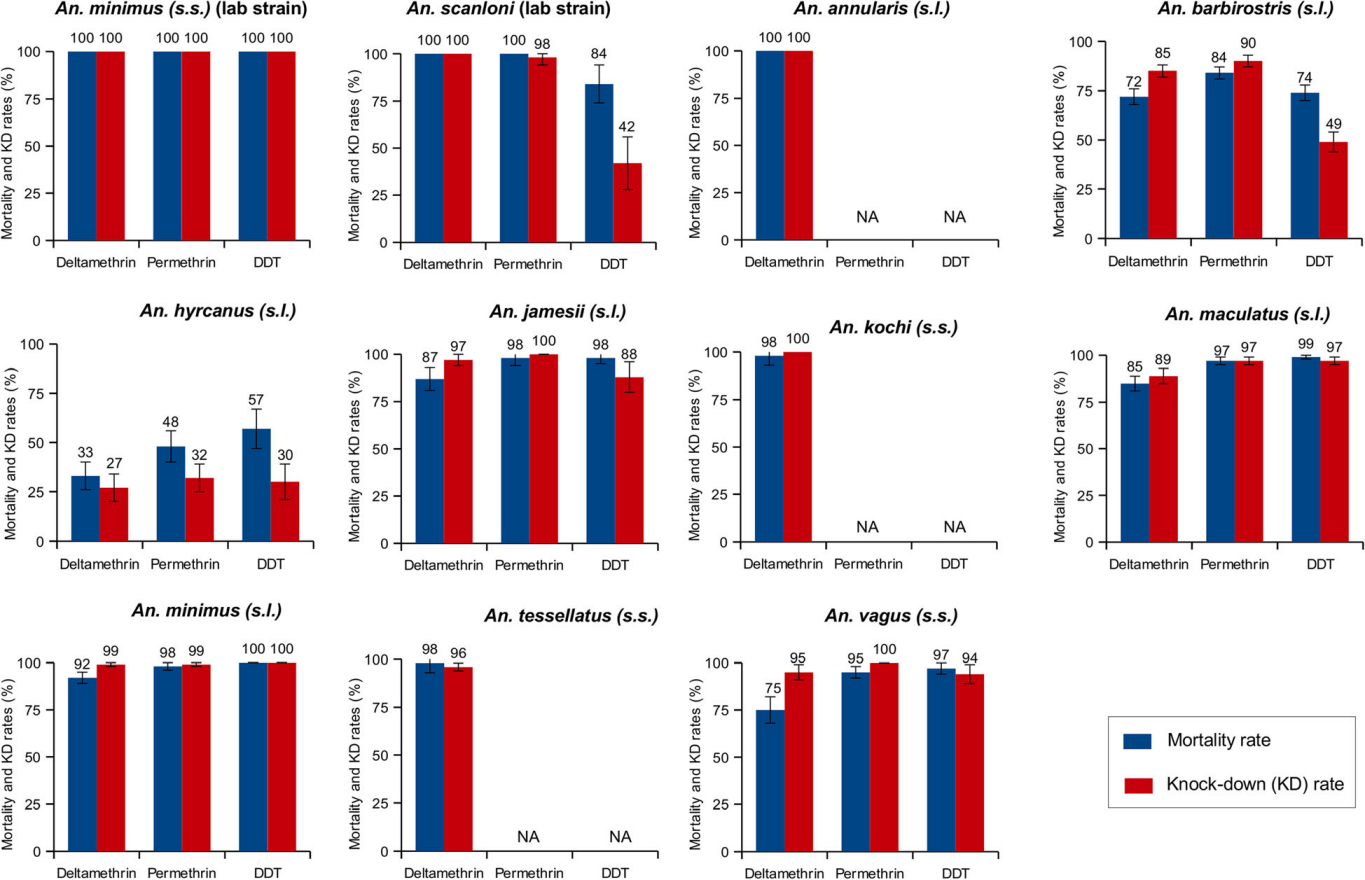
Mortality and knock-down (KD) rate determined following the WHO susceptibility test procedure for insecticide monitoring in malaria vectors. Alive female *Anopheles* were exposed during 1 h to insecticides (deltamethrin 0.05%, permethrin 0.75% and DDT 4%). KD rate was recorded at the end of the exposition period; the mortality rate was recorded after a 24 exposition period. *Abbreviation*: NA, not available

**Fig. 3:**
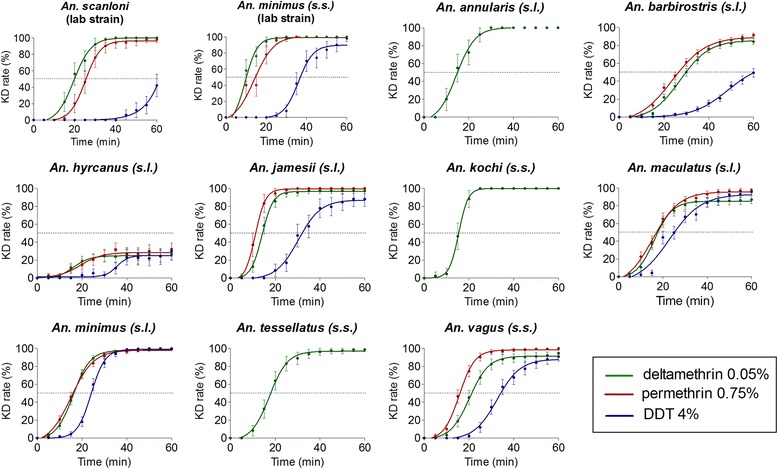
Kinetics of the knock-down (KD) rate during insecticide exposure. KD rate was recorded every five minutes during the exposition period to insecticide. *Dash-line* indicates a 50% KD rate

Mortality and knock-down (KD) rates are reported in Fig. 2 whereas the kinetic of KD (evolution of the KD rate over a 60 min observation period) is shown in Fig. 3 (data were analysed at the species complex or group when accurate identification at species level could not be done). Laboratory strain of *An. minimus* (*s.s*.) was susceptible to all insecticides whereas laboratory strain of *An. scanloni* was resistant to DDT (mortality of 84%, 42/50). The KDT50 of these strains were 10, 13 and 38 min for *An. minimus* (*s.s*.) and 20, 26 and 63 min for *An. scanloni* with deltamethrin, permethrin and DDT, respectively. Suspected resistance to deltamethrin was detected in wild caught *An. minimus* (*s.l*.) (92% mortality, 339/370). Resistance to deltamethrin and suspected resistance to permethrin were detected in *An. maculatus* (*s.l*.) (85 and 97% mortality, 239/280 and 257/264, respectively). *Anopheles barbirostris* (*s.l*.) and *An. hyrcanus* (*s.l*.) were resistant to all insecticides (mortality rates < 90%). *Anopheles vagus* was resistant to deltamethrin (75% mortality, 111/148) and suspected resistance to permethrin and DDT was also reported for this species (95 and 97% mortality, 169/178 and 123/133, respectively). Resistance to deltamethrin was detected in *An. jamesii* (*s.l*.) (87%, 97/111) whereas *An. annularis* (*s.l*.), *An. tessellatus* and *An. kochi* were susceptible to this pyrethroid insecticide. The susceptibility status of these latter species to permethrin and DDT could not be determined due to a very low sample size. Overall, the KDT50 varied from 10 to 131 min, from 13 to 116 min and 23 to 84 min for deltamethrin, permethrin and DDT, respectively (Tables 2, 3 and 4).

### Kdr detection

**Fig. 4:**
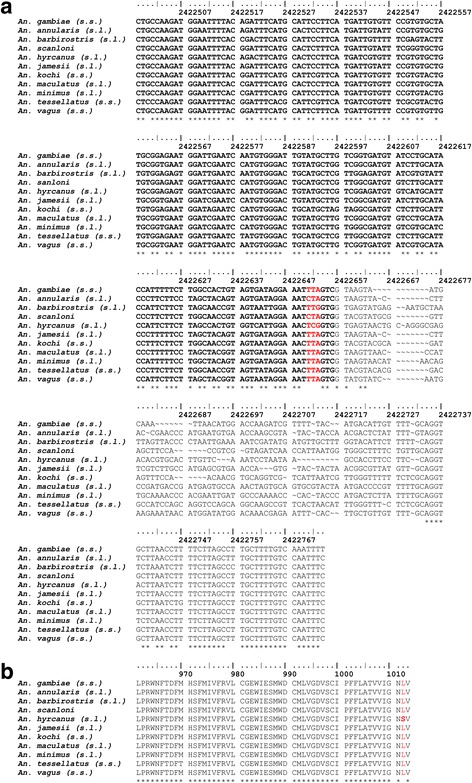
Molecular detection of the 1014 knock-down resistance (kdr) mutation on the voltage-gated sodium channel (VGSG) gene. **a** DNA sequence alignment of the fragment of VGSC gene encompassing nucleotides corresponding to the codon 1014 in various *Anopheles* species collected on the TMB. Bold character indicates the coding part of the DNA sequence (exon 20); codon 1014 is figured in red. **b** Consensus amino-acid sequence of the exon 20 determined for each anopheline taxa. The non-synonymous TCG polymorphism detected in *An. hyrcanus* (*s.l*.) is responsible for the L1014S kdr mutation; both heterozygous and homozygous mutations were detected. Other polymorphisms are either synonymous or located on non-coding part of the DNA sequence (GenBank Accession numbers KY677707–KY677716)

A PCR assay for kdr detection developed by Syaffrudin et al. [35] was first validated on ten species complexes collected along the TMB using two specimens per species (Fig. 4). The presence of kdr mutations was assessed in specimens surviving exposure to insecticide (post bioassays) to increase the chance to detect SNPs. The presence of kdr mutation was not investigated in *An. annularis* (*s.l*.), *An. kochi* and *An. tessellatus* considering the high mortality rates observed in bioassays (Table 5). The sequence alignment of the PCR product is presented in Fig. 4a. The non-synonymous L1014S mutation was found in *An. hyrcanus* (*s.l*.) (Fig. 4a, b) at both heterozygous (19%, 6/31) or homozygous state (10%, 3/31) (Table 5). No kdr mutations were detected in other anopheline species (Table 5).

**Table 5:** kdr mutation at position 1014 in the voltage-gated sodium channel (VGSG) gene

Taxa	*N* ^a^	1014 L^b^	L1014S^c^	
			Homozygous	Heterozygous
*An. annularis* (*s.l*.)	2	2	0	0
*An. barbirostris* (*s.l*.)	42	42	0	0
*An. hyrcanus* (*s.l*.)	31	22 (71%)	3 (10%)	6 (19%)
*An. jamesii* (*s.l*.)	55	55	0	0
*An. kochi*	2	2	0	0
*An. maculatus* (*s.l*.)	49	49	0	0
*An. minimus* (*s.l*.)	44	44	0	0
*An. tessellatus*	2	2	0	0
*An. vagus*	50	50	0	0

^a^ *N*, number of specimens genotyped

^b^ Number of specimens (%) carrying the wild-type genotype (homozygous 1014 L)

^c^ Number of specimens (%) carrying the homozygous or heterozygous 1014 L mutation

## Discussion

In this study, we assessed the susceptibility status of several anopheline taxa to three public health insecticides (deltamethrin, permethrin and DDT) along the TMB. To our knowledge, this is the first study reporting the susceptibility status of *Anopheles* mosquitoes to pesticides used for malaria control in this area. These data are important in the context of malaria elimination to select the most appropriate insecticides for vector control.

Bioassays were performed following the WHO guidelines for insecticide resistance monitoring in malaria vectors [37]. However, the WHO recommends the use of unfed 2–5 days old female *Anopheles* to assess the susceptibility of wild malaria vectors populations to insecticides. This implies to collect a sufficient number of larvae, which was not possible in the present study considering the difficulty of accessing to the collection sites. Since only adults were collected to run the bioassays, the mortality might be underestimated [49, 50]. Another limitation of the study was the identification of malaria vectors at the species level. Indeed most of *Anopheles* collected in the villages belong to complexes of sibling species i.e. groups of several species that are not distinguishable using conventional morphology criterions [51]. Previous data in the same study villages showed that *An. minimus* (*s.s*.) represents > 99% of the Minimus complex whereas *An. maculatus* (*s.s*.) and *An. sawadwongporni* were predominant within Maculatus group (unpublished data). Moreover, it was not always possible to reach the sample size recommended by the WHO for each test (80 mosquitoes for each insecticide and at least 20 mosquitoes for the control). Thus the interpretation of the results must take into account the sample size and the corresponding error bars.

In this study, suspected resistance to deltamethrin was detected in *An. minimus* (*s.s*.). Resistance to DDT was previously reported in Cambodia, Vietnam and Thailand [19, 20, 52] whereas resistance to pyrethroids (permethrin, alpha-cypermethrin and lambda-cyhalothrin) was only detected in Vietnam [19]. Few data have been collected so far on the susceptibility of *An. maculatus* (*s.s*.) and *An. sawadwongporni* to insecticides. In Thailand, resistance to DDT was documented by the Offices of Vector-Borne Diseases Control [52]. Overgaard et al. [21] later reported resistance of both sibling species to methyl-parathion in the Northern part of the country. No resistance to organophosphates, pyrethroids and DDT was detected however in neighbouring Malaysia [53, 54].

*Anopheles barbirostris* (*s.l*.) (confirmed secondary vector) and *An. hyrcanus* (*s.l*.) (suspected vector) were resistant to all insecticides. Pyrethroid resistance in *An. barbirostris* has only been described before in studies conducted in Indonesia [35, 55] and Sri Lanka [56]. Confirmed resistance to deltamethrin and suspected resistance to permethrin and DDT in *An. vagus* (suspected vector) are in agreement with a previous report showing a high level of resistance within the Hyrcanus group and in *An. vagus* [19, 23]. Unfortunately, it was not possible to generate data on the susceptibility of primary vectors belonging to the Dirus complex because of the low number of specimens collected.

Overall resistance to pyrethroids was reported in six out of the ten species complexes tested along TMB which suggests a strong selection pressure in the studied area. In northern Thailand, Overgaard et al. [21] previously demonstrated that resistance is likely to arise from the intense use of pesticides for agriculture (especially for organophosphates compounds, a class of insecticides that has never been used for vector control purposes). This must be taken into account by policy makers as additional use of insecticide (especially pyrethroids) for vector-control may lead to a rapid selection of a resistant phenotype as observed previously in Africa [8, 57]. Further efforts should be made to document the susceptibility status of malaria vectors to other classes of insecticides (such as carbamates, organophosphates and insect growth regulators) that could be used as an alternative to pyrethroids in the frame of resistance management strategies [58–60].

In this study, we briefly investigated the molecular mechanisms involved in pyrethroid and DDT resistance by using a PCR assay adapted from Syafruddin et al. [35]. The kdr mutation is known to induce a cross-resistance to both DDT and pyrethroids, and to be associated with an increase in the KDT50 values [6, 27]. Therefore we suspected this mechanism to be involved in the resistance of *An. hyrcanus* (*s.l*.) to the insecticides tested. We reported the occurrence of the L1014S kdr mutation in *An. hyrcanus* (*s.l*.) at a low frequency in the specimens surviving insecticide exposure. Specimens carrying a kdr mutation were further identified as *An. peditaeniatus* using molecular methods (data not shown) (Accession numbers: KY677698–KY677706). This finding confirms the previous report of the L1014S mutation in *An. peditaeniatus* populations from Southern Vietnam and Cambodia [23]. However, the occurrence of kdr mutations in other positions cannot be ruled out. For example, another kdr mutation (N1575Y) occurring within the domain VIII of the VGSG gene was found in *An. gambiae* from West Africa occurring in a V1014F haplotypic background [61]. Several authors have stressed previously that the absence of kdr mutations in most of the *Anopheles* species tested suggests that metabolic resistance is probably the main route of insecticide resistance in malaria vectors in Southeast Asia (i.e. *An. minimus* (*s.l*.), *An. maculatus* (*s.l*.) and *An. dirus* (*s.l*.)) [27, 31]. The metabolic basis of insecticide resistance in malaria vectors in the GMS remains largely unknown. The only metabolic mechanism described so far is the over-expression of two P450 isoforms (CYP6P7 and CYP6AA3) suspected to metabolise several pyrethroids [62] in a laboratory colony of deltamethrin-resistant *An. minimus* (*s.s*.) from Thailand [34, 63]. Further efforts should be made to decipher the molecular basis of insecticide resistance in Southeast Asian malaria vectors.

## Conclusion

Pyrethroid resistance seems to be widespread in *Anopheles* populations from the Thailand-Myanmar border. Documenting the susceptibility to other classes of insecticides is important in the framework of malaria control and elimination. Molecular basis of the resistance remains largely unknown in most of the Southeast Asian malaria vectors. Additional efforts should be made to identify molecular markers allowing the routine monitoring of insecticide resistance in this area.

## Abbreviations

DDT: Dichlorodiphenyltrichloroethane
DNA: Deoxyribonucleic acid
GMS: Greater Mekong Subregion
IRS: Indoor residual spraying
KD: Knock-down
kdr mutation: Knock-down resistance mutation
KDT50: Knock-down time 50
LLINs: Long-lasting insecticide-treated nets
TMB: Thailand-Myanmar border
VCRU: Vector Control Research Unit
VGSC: Voltage-gated sodium channel
WHO: World Health Organisation
